# An Optimized MRM-Based Workflow of the l-Arginine/Nitric Oxide Pathway Metabolites Revealed Disease- and Sex-Related Differences in the Cardiovascular Field

**DOI:** 10.3390/ijms23031136

**Published:** 2022-01-20

**Authors:** Benedetta Porro, Sonia Eligini, Edoardo Conte, Nicola Cosentino, Nicolò Capra, Viviana Cavalca, Cristina Banfi

**Affiliations:** Centro Cardiologico Monzino, IRCCS, 20138 Milan, Italy; benedetta.porro@cardiologicomonzino.it (B.P.); sonia.eligini@cardiologicomonzino.it (S.E.); edoardo.conte@cardiologicomonzino.it (E.C.); nicola.cosentino@cardiologicomonzino.it (N.C.); nicolo.capra@cardiologicomonzino.it (N.C.); viviana.cavalca@unimi.it (V.C.)

**Keywords:** l-homoarginine, l-arginine/nitric oxide metabolic pathway, targeted metabolomics, mass spectrometry, cardiovascular diseases, endothelial dysfunction

## Abstract

Clinical data indicate that low circulating l-homoarginine (HArg) concentrations are associated with cardiovascular (CV) disease, CV mortality, and all-cause mortality. A high number of LC-based analytical methods for the quantification of HArg, in combination with the l-arginine (Arg)-related pathway metabolites, have been reported. However, these methods usually consider a limited panel of analytes. Thus, in order to achieve a comprehensive picture of the Arg metabolism, we described an improved targeted metabolomic approach based on a multiple reaction monitoring (MRM) mass spectrometry method for the simultaneous quantification of the Arg/nitric oxide (NO) pathway metabolites. This methodology was then employed to quantify the plasma concentrations of these analytes in a cohort of individuals with different grades/types of coronary artery disease (CAD) in order to increase knowledge about the role of HArg and its associated metabolites in the CV field. Our results showed that the MRM method here implemented is suitable for the simultaneous assessment of a wide panel of amino acids involved in the Arg/NO metabolic pathway in plasma samples from patients with CV disease. Further, our findings highlighted an impairment of the Arg/NO metabolic pathway, and suggest a sex-dependent regulation of this metabolic route.

## 1. Introduction

l-homoarginine (HArg) is a naturally occurring non-proteinogenic amino acid synthesized from lysine mainly in the kidney [1] in a reaction catalyzed by l-arginine:glycine amidinotransferase (AGAT) [2], the first enzyme in the biosynthesis of creatine. HArg is present at low concentration in most body fluids [3,4], and its physiological role is still unknown. As HArg differs from l-arginine (Arg) by an additional methylene group, it competes as a weak substrate for endothelial nitric oxide (NO) synthase (NOS) [5], and it weakly inhibits arginase, the Arg-degrading enzyme [6], thus potentially enhancing NO formation by increasing Arg concentrations.

During the last decade, emerging evidence suggests that HArg exerts beneficial effects on endothelial and cardiac functions and vascular homeostasis [7,8,9]. An additional under-investigated, but potentially relevant, biological effect is the role of HArg as a specific, non-competitive inhibitor of the tissue-nonspecific alkaline phosphatase (TNAP), a rising predictor of cardiovascular (CV) risk [10]. Based on these premises, an increasing amount of evidence suggests that low HArg levels might represent a CV risk biomarker [11,12].

Indeed, clinical data indicate that low circulating HArg levels are associated with CV disease (CVD), CV mortality, and all-cause mortality [2,13,14,15,16,17,18]. In addition, several studies documented that lower than physiological plasma levels of HArg are associated with fatal CV events and mortality in patients subjected to coronary angiography, or with chronic kidney disease, heart failure, and stroke [14,18,19].

However, in order to recognize this molecule as a prognostic factor and therapeutic target in CVD, a better characterization of HArg homeostasis is needed.

Precise recognition of this compound in analytical methods that address the Arg/NO biosynthetic pathway is therefore important to fully understand the impact of the disease pathophysiology on this route. In the literature, a high number of liquid chromatography (LC)-based analytical methods for the quantification of HArg, in combination with the Arg-related pathway compounds, mainly NG,NG-dimethyl-l-arginine (asymmetric dimethylarginine, ADMA), and NG,N′G-dimethyl-l-arginine (symmetric dimethylarginine, SDMA) dimethyl arginines, have been reported [20,21,22,23,24,25,26]. However, as these methods are typically developed to solve specific biological issues, they consider a limited panel of analytes.

**Figure 1:**
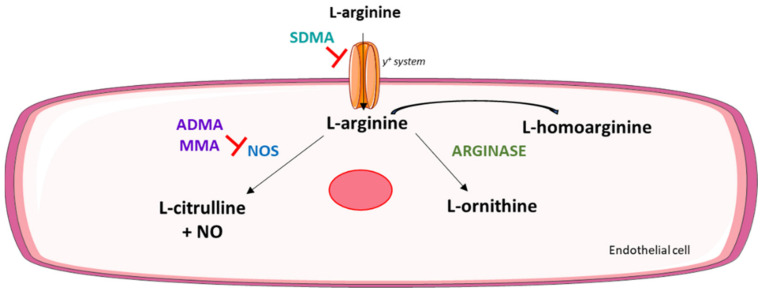
A schematic representation of the l-arginine/nitric oxide metabolic pathway in an endothelial cell.

Thus, in order to achieve a comprehensive picture of the Arg metabolism (Figure 1), this manuscript describes an improved targeted metabolomic approach based on a multiple reaction monitoring (MRM) mass spectrometry method for the simultaneous quantification of Arg, HArg, l-citrulline (Cit), l-ornithine (Orn), NG-monomethyl-l-arginine (MMA), ADMA, and SDMA. This methodology was then employed to quantify the plasma levels of these analytes in a cohort of individuals with different grades/types of coronary artery disease (CAD) in order to increase knowledge about the role of HArg and its associated metabolites in the CV field.

## 2. Results

### 2.1. Characteristics of the Study Participants 

In this study, we enrolled 104 subjects, 90 of whom underwent coronary computed tomography angiography (CCTA) at Centro Cardiologico Monzino IRCCS for suspected, but unknown, stable CAD. Based on the CCTA evaluation, patients were divided into three groups: no-CAD, non-obstructive (nonob)-CAD, and obstructive (ob)-CAD. Moreover, a group of 14 age- and sex-matched ST-segment elevated myocardial infarction (STEMI) patients was enrolled.

Demographic, clinical, and laboratory features of the study population are reported in Table 1. No-CAD patients were the youngest, although age was significantly different only in comparison with ob-CAD patients. As expected, STEMI patients showed the lowest HDL cholesterol and the highest triglyceride and basal glycemia levels compared to any other group.

The STEMI group also displayed the highest number of hypertensives, diabetics, and active smokers.

In Supplementary Table S1, the CCTA (for no-CAD, nonob-CAD, and ob-CAD) or OCT (for STEMI) atherosclerotic plaque features are reported. As regards the CCTA analyses, the coronary plaque volume in the whole population was 88.5 ± 128.2 mm^3^, and, as expected, was significantly higher in patients with ob-CAD when compared to those with nonob-CAD (*p* < 0.0001). Similarly, non-calcified plaque volume was higher in ob-CAD patients compared to that in nonob-CAD subjects. In 26 of 90 patients (28.6%), more than two high-risk plaque (HRP) features were identified; a high prevalence of HRP features was recorded among patients with ob-CAD (20 (62.5%) vs. 6 (23.1%), *p* < 0.0001 for ob-CAD vs. nonob-CAD patients).

As regards OCT plaque features identified in the STEMI group, a thrombus was present in 85.7% of the culprit lesions analyzed, and 71.4% of them showed a macrophage infiltration.

### 2.2. MRM-Based Analysis of the Arg/NO Pathway Metabolites

**Figure 2:**
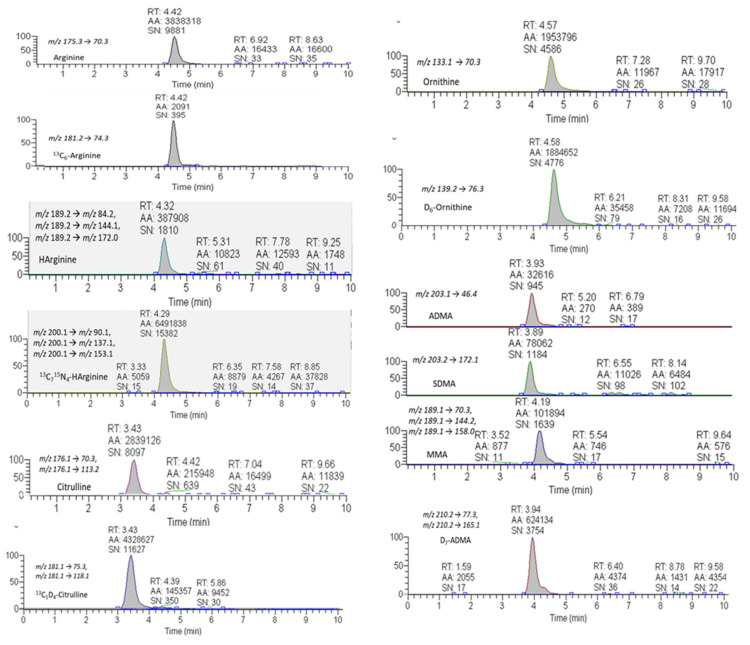
Representative chromatograms of HArg and its internal standard (^13^C_7_^15^N_4_-HArg), together with all the other metabolites involved in the Arg/NO metabolic pathway. The figure depicts the analysis of the pooled plasma (PP) added with a standard mix at a concentration of 2.5 μM.

The targeted metabolomic method for the simultaneous quantitative assessment of the Arg/NO pathway metabolites, here developed, was validated following the international FDA guidelines [27]. A representative chromatogram of HArg and of its internal standard ^13^C_7_^15^N_4_-HArg, together with all the other metabolites involved in the Arg/NO metabolic pathway measured in the pooled plasma (PP) sample, is shown in Figure 2. As depicted, the peaks related to HArg eluted at 4.3 min, in a region of the chromatogram free from any interfering background peaks.

The 8-point calibrator concentrations (0.156–20 μM) of the standard (HArg) prepared in solvent plotted against the ratio of the analyte/internal standard areas for five consecutive assays showed linear and reproducible curves with the following non-zero forced linear regression equation: y = (0.2683 ± 0.018)x + (0.0077 ± 0.005) (r^2^ = 0.999). Over the entire concentration range of the curve, the mean observed percentage deviation of the back-calculated concentrations was between −0.6% and +2.1%, with a coefficient of variation (CV) < 15%. As the human plasma HArg concentration seldom exceeds 5 μM, sample quantification was performed using a calibration curve in the range of 0.313–5 μM. Intra-assay and inter-assay imprecisions were < 15% for all quality controls (QC) tested; the LLOQ was 0.078 μM, while the LOD value was 0.039 μM. Detailed information is provided in Supplementary Table S2.

**Figure 3:**
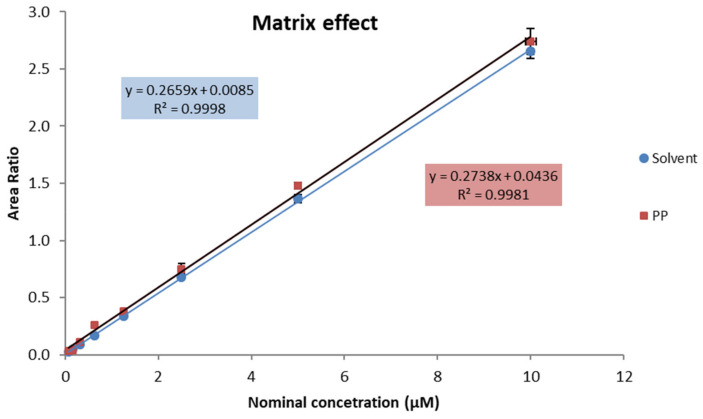
Representative curves of HArg standard dissolved in solvent (in blue) or in plasma pool sample (PP, in red).

Supplementary Table S3 shows the relative matrix effect (ME), extraction recovery (ER), and process efficiency (PE) of the method. To evaluate the matrix effects, calibration curves were also prepared in PP. The resulting curves were linear even if the slopes were slightly different from the slopes obtained in the solvent alone, indicating a minimal matrix effect for this analyte (Figure 3).

For this reason, the calibration functions obtained in the authentic matrix were used instead of those obtained in pure solvent. ER and PE values complied with the acceptability requirements, indicating the good reliability of the developed method.

HArg was highly stable in plasma at different temperatures of storage (from −20 °C, +4 °C, to room temperature (RT)), for at least 1 month (at −20 °C), and even throughout three freeze–thaw cycles (Supplementary Table S4).

The validation results for HArg are in accordance with those obtained for the other metabolites involved in the Arg/NO metabolic pathway [28]. In particular, the LOQ values make this method suitable for the evaluation of ADMA, SDMA, and MMA in samples containing relatively low concentrations of analytes with satisfactory precision, as documented by the intra- and inter-day CVs of less than 10%. The calibration functions in pure solvent were linear, with correlation coefficients greater than 0.99 for all compounds. As ADMA, SDMA, and MMA exhibited ME in plasma, the calibration functions obtained in the authentic matrix were used instead of those obtained in pure solvent.

### 2.3. Arg/NO Metabolic Pathway in Individuals with Different Grades of CAD

Table 2 reports the levels of all analytes involved in the Arg/NO metabolic pathway measured in the study cohort by means of the targeted metabolomic method here implemented. All the metabolites were similar among no-CAD, nonob-CAD, and ob-CAD patients, whilst in STEMI patients we assessed a significant derangement of this metabolic route when compared to any other study group. Specifically, in STEMI patients both substrates Arg and HArg, and the NOS product Cit, significantly decreased, whilst the arginase product Orn and the competitive inhibitor of Arg transport, SDMA, increased.

As regards clinical parameters, age- and sex-adjusted HArg levels were inversely associated with platelet count (r = −0.547, *p* = 0.007) only in the nonob-CAD group. In STEMI patients, instead, HArg was significantly correlated with red blood cell (RBC) count (r = 0.723, *p* = 0.008) and its related parameters hemoglobin (Hb), and hematocrit (Hct) (r = 0.586, *p* = 0.045; r = 0.583, *p* = 0.046, respectively). 

Further, among the Arg/NO pathway metabolites, the correlation analysis in the overall population revealed a positive association between HArg and Arg, the primary substrate of NOS (r = 0.260, *p* = 0.007). This association was confirmed also when our study group was divided according to the diagnosis (r = 0.469, *p* = 0.024; r = 0.398, *p* = 0.018, in nonob-CAD and ob-CAD, respectively). In addition, we found a negative association between HArg and ADMA, the major endogenous inhibitor of NOS (r = −0.485, *p* < 0.0001), in the entire study group. This result was reinforced by the analysis in the patient subgroups, which showed a negative correlation between HArg and the competitor of Arg transport SDMA (r = −0.525, *p* = 0.002; r = −0.339, *p* = 0.0465; r = −0.632, *p* = 0.0275, in no-CAD, ob-CAD, and STEMI subjects, respectively), and a positive correlation between HArg and Arg to SDMA ratio, which is considered an indicator of Arg uptake by the cell (r = 0.455, *p* = 0.01; r = 0.414, *p* = 0.0496; r = 0.492, *p* = 0.003, in no-CAD, nonob-CAD, and ob-CAD, respectively).

### 2.4. The Effect of Sex on the Arg/NO Metabolic Pathway

**Figure 4:**
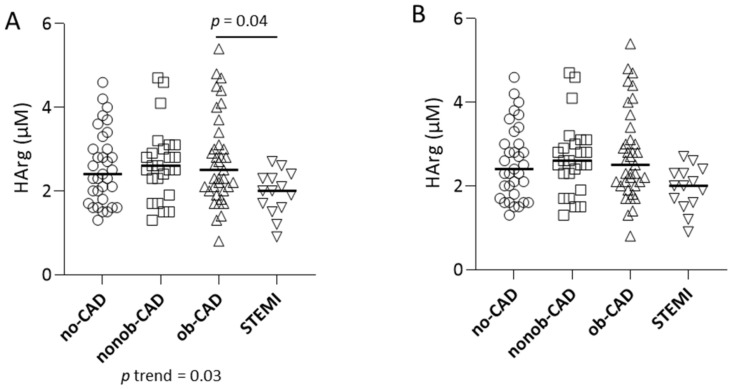
(**A**) Levels of HArg in plasma from no-CAD (ο), nonob-CAD (□), ob-CAD (△), and STEMI (▽) patients (raw data); (**B**) levels of HArg in plasma from no-CAD, nonob-CAD, ob-CAD, and STEMI patients, adjusted for age and sex. Data are represented using a scatter dot plot; horizontal lines show median value.

The univariate analysis of HArg plasma levels revealed a reduction in the analyte levels in patients with the acute manifestation of CAD (STEMI), with no differences based on the grade of coronary artery stenosis (Figure 4A). However, after the adjustment for confounders (age and sex), this difference became non-significant (Figure 4B). Of interest, the difference in the levels of Arg and SDMA, as well as the reduction of the ratios HArg/SDMA, HArg/ADMA + MMA + SDMA, and HArg/Orn in the STEMI group, remained significant even after adjustment for age and sex (Table 2), reinforcing the evidence of an effect of the acute disease on the Arg/NO metabolic pathway.

**Figure 5:**
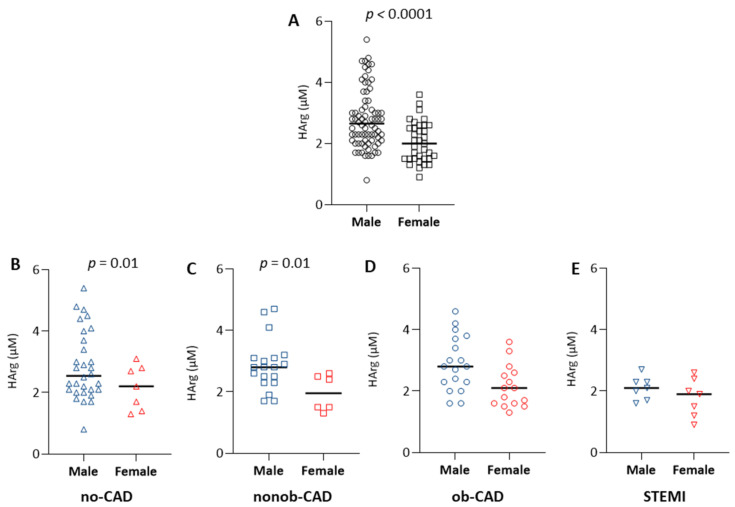
(**A**) Levels of HArg in plasma from male and female (raw data), independent of the disease condition. Levels of HArg in plasma from (**B**) no-CAD, (△), (**C**) nonob-CAD (□), (**D**) ob-CAD (ο), and (**E**) STEMI (▽) patients, based on sex. Data are represented using a scatter dot plot; horizontal lines show the median value.

Related to this, when we divided our study group based on sex, we found increased HArg levels in males compared to females (Figure 5A), independent of the disease condition. This difference was maintained even after adjustment for age (*p* = 0.05).

By stratifying our data based on diagnosis, we found that this sex-based difference was evident only in no-CAD and nonob-CAD patients (Figure 5B,C), whereas, when CAD becomes evident, HArg levels in males become similar to those measured in the female group (Figure 5D,E).

**Figure 6:**
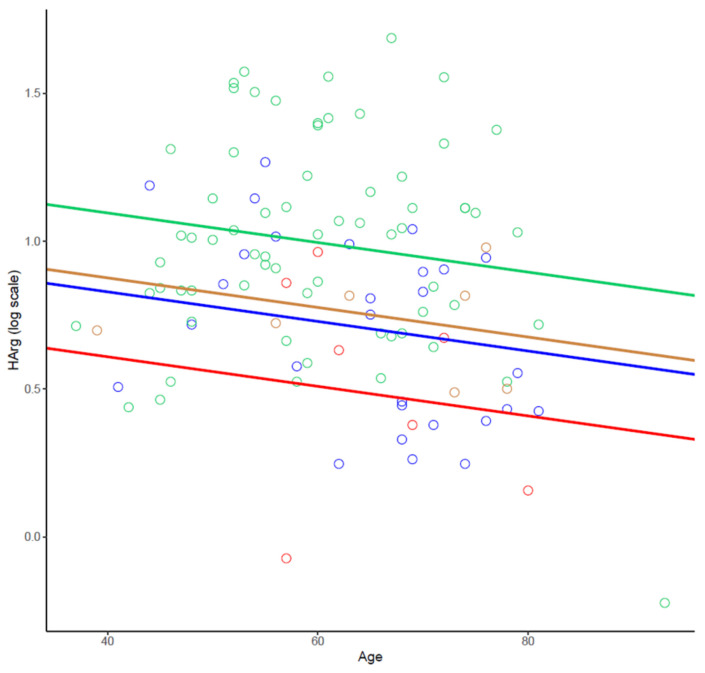
Covariance analysis of HArg in no-STEMI (no-CAD, nonob-CAD, and ob-CAD) and STEMI cohort, based on sex. Female no-STEMI as blue circles, female STEMI as red circles, male no-STEMI as green circles, and male STEMI as brown circles. Data are represented as individual values.

In order to substantiate our results, we performed a covariance analysis grouping no-CAD, nonob-CAD, and ob-CAD patients in the no-STEMI cohort, and compared them with the STEMI one. As reported in Figure 6, the male STEMI group (blue colored) showed a decrease in HArg levels of 20% compared to no-STEMI males. Similarly, in the female cohort, in which no-STEMI patients (brown colored) had HArg levels comparable to those of STEMI males, we found a significant decrease in the acute manifestation of the disease (equal to 30%), confirming the presence of a pathology-based effect (*p* = 0.02).

## 3. Discussion

The optimized, sensitive, specific, and selective targeted metabolomic method (based on multiple reaction monitoring (MRM)) here presented is suitable for the simultaneous assessment of a wide panel of amino acids involved in the Arg/NO metabolic pathway in plasma samples from both healthy subjects and patients with different CAD manifestations.

In the last few years, substantial research has been undertaken to clarify the role of molecules involved in the Arg/NO metabolic pathway in specific physiological processes. Many LC-based analytical methods have been developed [20,21,22,23,24,25,26,29], but, as these approaches are typically focused on specific research questions, they consider a relatively limited panel of analytes.

The use of MRM-based approaches provides the advantage of rapidly scanning over multiple (very narrow) mass windows, thus acquiring traces of multiple fragment ion masses from one or more precursor ions simultaneously. Advantageously, with respect to other strategies [30], the utilization of the stable-labeled isotopes of each metabolite as internal standard guarantees the almost identical physico-chemical properties of the target molecule, and allows for monitoring of every sample preparation step. Further, without the need for a solid phase extraction procedure, this LC-tandem mass spectrometry (MS)-based method minimizes sample manipulation, avoiding process losses. To date, almost all published methods show some pitfalls regarding certain analytes of interest. Indeed, with the exception of the assay described by Zhang and Kaye [31], which is currently the most comprehensive test in terms of the compounds considered, to the best of our knowledge no published assay considers the full panel of the Arg/NO metabolic pathway metabolites. Indeed, the approach of Zhang and Kaye does not allow for the characterization of plasma HArg, as its internal standard was used at a concentration 100-fold greater than the normal physiological concentration range.

Van Dyk and collaborators developed a method similar to that implemented by us [32]. However, their analytical approach included an evaporation to dryness step, resulting in a possible loss of sample, which could not be correctly normalized because the authors only used D_6_-ADMA as internal standard for all seven analytes tested.

NO is a well-known key mediator of homeostatic processes and host defense mechanisms [33]. The diminished bioavailability and the dysfunctional metabolism of Arg, the biological precursor of NO, are associated with increased CV risk and CAD development [34]. Furthermore, in recent years, two separate large cohorts assessed the association between low circulating HArg concentrations and CVD outcomes and mortality [14].

Thus, in our work, we applied an MRM method to screen Arg metabolism in a cohort of CAD patients with different grades of stenosis, from individuals without any obstruction in the main coronary vessels to patients with acute myocardial infarction, in order to achieve the most complete overview of the behavior of the main metabolites involved in this metabolic pathway.

Indeed, ADMA, SDMA, and HArg are non-proteinogenic amino acids structurally related to Arg, and they influence NO formation. Specifically, HArg acts as an alternative substrate for NOS and inhibits arginase, thus increasing NO formation [35]. ADMA, on the contrary, is an endogenous inhibitor of NOS [36], whereas its structural isomer SDMA does not directly interfere with NOS, but, through the inhibition of the tubular Arg absorption in the kidney [37] and by the blockage of the y+ transporter, which mediates the intracellular Arg uptake [38], it can indirectly affect NOS. Due to their effects on NO, both dimethylarginines, ADMA and SDMA, are involved in endothelial dysfunction [37,39], oxidative stress [40], and atherosclerosis [41], and are both independently associated not only with CVD but also with all-cause mortality [42].

Finally, Orn is also a non-proteinogenic amino acid that plays a role in the urea cycle. It is produced from Arg by the enzyme arginase, which has been shown to be a crucial mediator of endothelial dysfunction in several pathologic states [43,44,45].

Consistent with data from the literature in various CVD settings [46,47,48,49], in our study population we also found reduced levels of Arg and HArg and increased levels of SDMA and Orn in patients with STEMI. The reduction in the ratios HArg/SDMA, HArg/ADMA + MMA + SDMA, and HArg/Orn in the STEMI group remains significant even after adjustment for age and sex, suggesting a decrease in NO production and reinforcing the evidence of an effect of the acute disease on HArg metabolism.

Aging is considered the strongest independent risk factor for CAD as it is associated with increased oxidative stress, inflammation, and shifts in gene expression that contribute to increased vascular stiffness, endothelial dysfunction, and thrombogenicity [50,51]. In accordance with this finding, in our population subjects with a diagnosis of CAD were older than those in the no-CAD group. Alongside age, male gender is considered the other major contributor in increasing CAD risk development [50,52]. For this reason, both these confounders were considered in the analysis of results. Nevertheless, all the differences in the levels of the Arg/NO pathway metabolites evidenced in the STEMI group remain significant even after the adjustment for confounders, with the exception of HArg.

Sex-based differences are also known to exist in multiple circulating biomarkers associated with cardiovascular risk and, between them, those involved in endothelial dysfunction [53,54], suggesting possible diversities also in pathophysiological mechanisms contributing to CVD.

To the best of our knowledge, there are no studies on sex differences in Arg/NO metabolic pathway during acute cardiovascular events.

Interestingly, the analysis of HArg levels revealed higher concentrations of the analyte in males than in females until the atherosclerotic disease became manifest (CAD group), and then equalized between sexes in STEMI. Indeed, the presence of an acute coronary syndrome resulted in a reduction of HArg both in males and females, suggesting the presence of a pathology-based effect. This finding was also confirmed by the covariance analysis that revealed how the HArg behavior in the male STEMI group was comparable to that showed in the female no-STEMI cohort. We can only propose some hypotheses on this issue. For example, the estrogen regulation of l-arginine:glycine amidinotransferase (AGAT), an enzyme that catalyzes the committed step in creatine biosynthesis, was firstly demonstrated in chick liver [55]. The sex-dependent activity of the enzyme involved in HArg biosynthesis, combined with the association between low HArg levels and the increased probability of fatal and non-fatal cardiovascular events [56,57,58], could explain the lack of sex difference in the levels of this metabolite in the STEMI cohort. Further, a strong correlation between HArg deficiency and heart disease severity has also been demonstrated in experimental animal models [2,7,8,59]. However, as data on the underlying molecular mechanisms and signal transduction pathways involved in HArg and in the associated AGAT enzyme metabolism are still very limited, further studies on larger cohorts are needed.

In this study, we found no differences in the Arg/NO pathway metabolites in patients with different degrees of CAD. This result appears to be in contrast with some studies present in the literature [60,61,62]. For instance, Chen and coworkers evidenced a reduction in nitrate and nitrite (NOx), Arg, ADMA, and Arg/ADMA ratio in plasma from patients with syndrome X compared with control subjects [61]. Also, Piatti et al. and our group published similar results in the same clinical setting [60,62] suggesting the presence of an alteration of the Arg/NO pathway that could be responsible for the abnormal vascular reactivity characterizing patients with microvascular bed alterations.

The fact that in our population we saw no difference among CAD patients and the no-CAD group could be related to the use of CCTA. Even if this technique is recommended by the current guidelines as the first line diagnostic tool for the identification of stenosis in the main epicardial tree [63], it is unable to analyze the microcirculatory bed, preventing us from highlighting possible alterations in the microcirculation. This can be considered one of the limitations of this study, which may prevent us from distinguishing between truly healthy individuals and individuals with altered microcirculation. As in our work, the no-CAD group is composed of individuals with, in some cases, a positive stress test or a chest pain episode, we cannot exclude the presence of microcirculatory alterations in these subjects, which we know be linked to a derangement of the Arg/NO pathway. 

## 4. Conclusions

In conclusion, our results, although obtained on a small population size by means of an optimized MRM approach, highlight an impairment of the Arg/NO metabolic pathway, and suggest a sex-dependent regulation of this metabolic route.

## 5. Materials and Methods

### 5.1. Chemicals and Reagents

^13^C_6_-Arg, D_6_-ornitine, ^13^C_1_D_4_-citrulline, ^13^C_7_^15^N_4_-HArg, and D_7_-ADMA were purchased from Cambridge Isotope Laboratories, Inc. (Andover, MA, USA). Arg, HArg, Orn, Cit, ADMA, SDMA, and MMA were purchased from Merck Millipore Ltd. (Cork, Ireland). Purified water was obtained from a Milli-Q^®^ Integral system; all other chromatography-grade chemicals were obtained from Merck Millipore Ltd. (Cork, Ireland).

### 5.2. Study Population

In this study, we enrolled 90 consecutive patients who underwent coronary computed tomography angiography (CCTA), between March 2016 and February 2018, for suspected but unknown stable CAD. Based on CCTA evaluation, patients were divided into three groups: no-CAD, non-obstructive (nonob)-CAD, and obstructive (ob)-CAD. In detail, the no-CAD group showed the absence of any plaque in the coronary tree (0% stenosis and no luminal irregularities); the nonob-CAD group presented limited atherosclerotic disease evidenced by a stenosis < 50%; and the ob-CAD group displayed an atherosclerotic disease with a stenosis ≥ 50%. Moreover, a group of unstable CAD patients was enrolled. Fourteen age- and sex-matched ST-segment elevated myocardial infarction (STEMI) patients undergoing emergent/urgent coronary angiography as their first manifestation of ischemic heart disease were enrolled. STEMI was defined as prolonged chest pain (>30 min) with pain onset < 12 h, ST-segment elevation > 0.2 mV in at least two contiguous leads in the initial electrocardiogram (ECG), and elevated serum troponin I levels. The presence of traditional CV risk factors such as diabetes mellitus (fasting glucose level of 126 mg/dL or higher and/or the need for insulin or oral hypoglycemic agents), hypercholesterolemia (total cholesterol level > 200 mg/dL or treatment with lipid-lowering drugs), hypertension, smoking attitude, and family history of CAD, were further investigated for all participants. This study was carried out in accordance with the Declaration of Helsinki and approved by the local ethics research committee of Centro Cardiologico Monzino. Written informed consent to participate was obtained from all subjects.

### 5.3. Sample Collection, CCTA and OCT Protocol, Images Reconstruction, and Analysis 

Peripheral blood samples from all participants were drawn into EDTA-containing tubes and kept on ice. For the CCTA cohort, the blood sample was obtained before the CCTA procedure, while for the STEMI group sample collection was performed before coronary angiography. Whole blood was centrifuged at 1700× *g* for 10 min at 4 °C to obtain plasma and plasma aliquots were stored at −80 °C until analysis. For the validation procedure, a pooled plasma (PP) sample was obtained from the plasma of different volunteers mixed together (please see the Supplementary Materials). For samples preparation, 50 μL of plasma was mixed with 400 μL of acetonitrile:methanol (50:50, v/v) after adding 50 μL of the internal standard mixture containing ^13^C_7_^15^N_4_-HArg 2 μM (10-fold diluted plasma) and the other internal standards related to the Arg/NO pathway as previously reported [28]. Precipitated proteins were separated by centrifugation at 12,000× *g* for 20 min at 4 °C and supernatants were used for analysis.

As regards the CCTA group, patients were treated before a CT scan with an intravenous beta-blocker (Metoprolol up to 20 mg) to optimize heart rate and with a standard dose of sublingual nitrates as previously reported [64,65].

The detailed descriptions of the CCTA and OCT protocol, images reconstruction, and analysis are reported elsewhere [64,66,67,68,69,70,71].

### 5.4. Targeted Metabolomics of the Arg/NO Pathway by Means of an MRM-Based Approach

The chromatographic separation was conducted using an Accela HPLC System (Thermo Fisher Scientific, San Jose, CA, USA) equipped with a Luna HILIC analytical column (50 × 2.0 mm, 3 μm; Phenomenex, Torrance, CA, USA), maintained at 30 °C. Samples (10 μL) were eluted with a gradient of mobile phase (Supplementary Table S5) during a total run time of 25 min. A triple-quadrupole TSQ Quantum Access (Thermo Fisher Scientific) mass spectrometer equipped with an electrospray ionization (ESI) interface operated in positive mode was used for mass spectrometric analysis. The HArg and its internal standard were detected by MS/MS using multiple reaction monitoring (MRM) by monitoring the transitions m/z 189.2 → m/z 84.2, m/z 189.2 → m/z 144.1, and m/z 189.2 → m/z 172.0 (for HArg) and m/z 200.1 → m/z 90.1, m/z 200.1 → m/z 137.1, and m/z 200.1 → m/z 153.1 (for ^13^C_7_^15^N_4_-HArg). The transitions referred to for Arg, ^13^C_6_-Arg, ADMA, SDMA, MMA, D_7_-ADMA, Orn, D_6_-Orn, Cit, and ^13^C_1_-D_4_-Cit have been previously reported [27]. Briefly, m/z 175.3 → 70.3 for Arg; m/z 181.2 → 74.3 for ^13^C_6_-Arg; m/z 203.1 → 46.4 for ADMA; m/z 203.2 → 172.1 for SDMA; m/z 189.1 → 70.3, m/z 189.1 → 144.2, and m/z 189.1 → 158.0 for MMA; m/z 210.2 → 77.3 and m/z 210.2 → 165.1 for D_7_-ADMA; m/z 133.1 → 70.3 for Orn; m/z 139.2 → 76.3 for D_6_-Orn; m/z 176.1 → 70.3 and m/z 176.1 → 113.2 for Cit; and m/z 181.1 → 75.3 and m/z 181.1 → 118.1 for ^13^C_1_-D_4_-Cit.

The operating conditions for MS analysis were as follows: spray voltage, 2500 V; capillary temperature and voltage, 260 °C and 35 V, respectively; and sheath gas and auxiliary gas flow, 25 and 20 arbitrary units, respectively. Xcalibur^®^ software, version 2.0 (Thermo Fisher Scientific, Waltham, MA, USA), was used for system control, data acquisition, and processing. A detailed description of the LC-MS/MS method used for the target metabolomics analysis of the analytes involved in the Arg/NO biosynthetic pathway is reported elsewhere [28].The method validation for HArg analysis was based on the guidelines of the U.S. Food and Drug Administration [27] and Matuszewski [72], including the evaluation of imprecision, linearity range, lower limit of quantification (LLOQ), limit of detection (LOD), relative matrix effect (ME), extraction recovery (ER), and process efficiency (PE) and sample stability. Validation process details are reported in the Supplementary Materials.

### 5.5. Statistical Analysis

Continuous variables were presented as the mean ± SD or as the median with interquartile range [IQR: 25°–75°], if more appropriate. Continuous variables normally distributed were compared using the Student’s *t*-test for independent samples. When the variable distribution was not normal, Mann–Whitney U tests for independent samples were used. Variables with positively skewed distributions were log-transformed before analysis. The proportion of the categorical variables was compared using an χ2 analysis or Fisher exact test, as appropriate. A *p*-value < 0.05 was considered statistically significant. Analysis of covariance (ANCOVA) was applied to examine if the differences in the mean HArg values were related to the sex- and/or diagnosis-based effects. Statistical analysis and graphics were produced with MedCalc (version 11.6.1.0, Med-Calc Software; 1993–2011) and by the SAS v. 9.4 statistical package (SAS inc. Cary, NC, USA).

## Figures and Tables

**Table 1:** Demographic and clinical characteristics of the study population.

	All Patients (*n* = 104)	No-CAD (*n* = 33)	Nonob-CAD (*n* = 25)	Ob-CAD (*n* = 32)	STEMI (*n* = 14)	ANOVA *p*-Value
Demographic and clinical characteristics						
Age, years	61.6 ± 10.9	57.1 ± 11.5	61.3 ± 10.7	65.5 ± 9.7 *	65.4 ± 11.3	0.01
Male, *n* (%)	69 (66.3)	18 (54.5)	19 (76.0)	24 (75)	7 (50.0)	0.12
BMI, kg/m^2^	25.9 ± 3.9	25.5 ± 4.5 ^◊^	25.3 ± 3.8 ^◊^	25.7 ± 3.3 ^◊^	29.1 ± 3.0	*0.01*
WBC, 10^3^/μL	7.5 ± 2.2	7.8 ± 2.5	7.4 ± 2.2	7.1 ± 2.2	7.9 ± 2.2	0.57
Platelet, 10^3^/μL	221.2 ± 64.1	242.8 ± 70.0	220.9 ± 61.9	205.5 ± 52.0	214.8 ± 80.7	0.13
MPV, fL	10.4 ± 1.3	10.5 ± 0.8	10.3 ± 1.4	10.3 ± 2.0	10.7 ± 0.7	0.78
RBC, 10^6^/μL	4.7 ± 0.7	4.7 ± 0.5	4.8 ± 0.6	4.7 ± 0.8	4.3 ± 0.8	0.15
Hb, g/dL	14.2 [13.1–15.1]	13.7 [13–14.6]	14.4 [13.3–15.2] ^◊^	14.4 [13.4–15.4] ^◊^	12.8 [11.5–13.5]	0.004
Hct, %	41.0 ± 5.0	40.4 ± 3.2	41.6 ± 4.6	41.9 ± 6.2 ^◊^	37.6 ± 6.5	0.05
MCV, fL	87.6 ± 4.8	86.4 ± 5.5	87.6 ± 4.6	88.4 ± 3.1	87.8 ± 6.3	0.40
MCH, pg	30.2 ± 2.0	29.5 ± 2.1	30.2 ± 1.9	30.7 ± 1.2	29.9 ± 2.7	0.09
MCHC, %	34.4 ± 1.0	34.1 ± 1	34.5 ± 1.0	34.8 ± 0.8 *	34.0 ± 1.1	0.01
RDW-CV, %	13.3 ± 1.0	13.2 ± 0.9	13.2 ± 0.9	13.1 ± 0.9 ^◊^	14.0 ± 1.6	0.04
RDW-SD, fL	41.6 ± 2.9	40.8 ± 2.1 ^◊^	41.4 ± 2.8	41.6 ± 3.2	43.5 ± 2.6	0.02
Total cholesterol, mg/dL	198.6 ± 39.6	196.3 ± 35.2	199.5 ± 38.2	206.8 ± 36.1	199.6 ± 43.4	0.72
LDL cholesterol, mg/dL	119.9 ± 35.4	116.9 ± 29.1	119.9 ± 34.3	129.3 ± 31.4	128.1 ± 37.0	0.40
HDL cholesterol, mg/dL	56.0 ± 17.2	60.2 ± 14.8 ^◊^	57.4 ± 15.5 ^◊^	54.8 ± 12.3 ^◊^	42.9 ± 13.3	0.002
Triglycerides, mg/dL	115.0 ± 55.9	97.6 ± 47.6 ^◊^	106.6 ± 48.9 ^◊^	113.4 ± 49.5 ^◊^	178.9 ± 55.4	<0.0001
Basal glucose, mg/dL	103.8 ± 22.6	103.3 ± 19.7 ^◊^	98.6 ± 15.3 ^◊^	100.8 ± 16.2 ^◊^	131.4 ± 34.3	<0.0001
Hypertension, *n* (%)	19 (18.3)	14 (42.4) ^◊^	1 (4.0) *^,^°^,^^◊^	17 (53.1) ^◊^	12 (85.7)	<0.0001
Family history of CVD, *n* (%)	22 (21.1)	10 (30.3)	3 (12.0)	11 (34.4) ^◊^	8 (57.1)	0.03
Dyslipidaemia, *n* (%)	50 (48.1)	13 (39.4)	14 (56.0)	10 (31.2)	6 (42.8)	0.30
Diabetes, *n* (%)	7 (6.7)	2 (6.1) ^◊^	0 (0) ^◊^	2 (6.2) ^◊^	5 (35.7)	0.001
Active smokers, *n* (%)	13 (12.5)	5 (15.2) ^◊^	1 (4.0) ^◊^	6 (18.7) ^◊^	10 (71.4)	<0.0001
*Pharmacological treatments*						
β-blockers, *n* (%)	25 (24.0)	16 (48.5) ^◊^	6 (24.0)	20 (62.5) °^,^^◊^	2 (14.3)	0.003
ACE-inhibitors, *n* (%)	21 (20.2)	6 (18.2)	4 (16.0)	6 (18.7)	4 (28.6)	0.79
Aspirin, *n* (%)	28 (26.9)	8 (24.4)	4 (16.0)	8 (25)	7 (50.0)	0.13
Statins, *n* (%)	31 (29.8)	7 (21.1)	12 (48.0)	10 (31.2)	4 (28.6)	0.16

BMI = Body mass index; CVD = cardiovascular disease; Hb = hemoglobin; Hct = hematocrit; HDL = high-density lipoprotein; HQ = high quartile; LDL = low-density lipoprotein; MCH = mean corpuscular hemoglobin; MCHC = mean corpuscular hemoglobin concentration; MCV = mean corpuscular volume; MPV = mean platelet volume; RBC = red blood cell; RDW-CV = red blood cell distribution width-coefficient of variation; RDW-SD = red blood cell distribution width-standard deviation; WBC = white blood cell. * *p* < 0.05 vs. no-CAD; ° *p* < 0.05 vs. nonob-CAD; ^◊^ *p* < 0.05 vs. STEMI.

**Table 2:** Levels of all analytes involved in the Arg/NO metabolic pathway measured in the study population.

	No-CAD (*n* = 33)	Nonob-CAD (*n* = 25)	Ob-CAD (*n* = 32)	STEMI (*n* = 14)	*p*-Value Not Adjusted	*p*-Value Adjusted for Age	*p*-Value Adjusted for Age and Sex
Arg, μM	87.8 ± 21.8 *^◊^*	92.0 ± 18.0 *^◊^*	90.4 ± 22.4	62.0 ± 23.3	0.0002	0.0003	<0.0001
Cit, μM	33.2 ± 6.3	32.5 ± 8.4	33.8 ± 7.4	27.4 ± 9.3	0.70	0.06	0.048
Orn, μM	68.0 [61.7;77.2] *^◊^*	61.5 [54.1;74.6] *^◊^*	69.8 [62.3;77.5] *^◊^*	99.9 [69.3;105.1]	0.006	0.003	0.003
Harg, μM	2.4 [1.8;3.0]	2.6 [2.3;3.0]	2.5 [2.0;3.1]	2.0 [1.6;2.3]	0.03	0.04	0.16
ADMA, μM	0.36 ± 0.1	0.37 ± 0.1	0.36 ± 0.1	0.37 ± 0.1	0.95	0.81	0.87
SDMA, μM	0.7 [0.6;0.9] *^◊^*	0.8 [0.6;1.0] *^◊^*	0.8 [0.6;1.1] *^◊^*	1.0 [0.9;1.3]	0.01	0.002	0.002
MMA, μM	0.1 [0.1;0.1]	0.1 [0.1;0.1]	0.1 [0.1;0.1]	0.1 [0.1;0.1]	0.05	0.18	0.20
Arg/Orn + Cit	0.9 ± 0.2	0.9 ± 0.2 *^◊^*	0.9 ± 0.3 *^◊^*	0.5 ± 0.2	<0.0001	<0.0001	<0.0001
Arg/ADMA + MMA	188.3 ± 45.8 *^◊^*	193.2 ± 50.0 *^◊^*	194.5 ± 59.5 *^◊^*	125.7 ± 41.6	<0.0001	0.0002	0.0002
Arg/SDMA	119.4 ± 38.8 *^◊^*	124.6 ± 54.8 *^◊^*	118.6 ± 50.1 *^◊^*	60.0 ± 32.5	<0.0001	0.0005	<0.0001
Arg/ADMA + MMA + SDMA	71.9 ± 19.5 *^◊^*	73.6 ± 23.1 *^◊^*	71.9 ± 24.5 *^◊^*	39.8 ± 19.1	<0.0001	<0.0001	<0.0001
Orn/Cit	2.0 [1.9;2.3] *^◊^*	1.9 [1.7;2.4] *^◊^*	2.1 [1.7;2.5] *^◊^*	3.1 [2.8;3.6]	<0.0001	<0.0001	<0.0001
Harg/SDMA	3.4 [1.7;5.2] *^◊^*	3.2 [2.6;4.7] *^◊^*	3.2 [2.0;5.0] *^◊^*	2.1 [1.2;2.6]	0.004	0.002	0.004
Harg/ADMA + MMA + SDMA	2.1 [1.2;3.0] *^◊^*	2.1 [1.7;2.5] *^◊^*	2.1 [1.3;2.9] *^◊^*	1.5 [0.9;1.6]	0.006	0.003	0.009
HArg/ADMA + MMA	5.3 [3.9;7.1]	5.6 [4.3;6.3]	5.3 [4.1;7.1]	4.2 [2.6;5.5]	0.097	0.04	0.19
Harg/Orn	0.04 [0.05;0.05] *^◊^*	0.04 [0.03;0.05] *^◊^*	0.04 [0.03;0.05] *^◊^*	0.02 [0.02;0.03]	0.001	0.003	0.001
Harg/Orn + Cit	0.02 [0.02;0.03]	0.03 [0.02;0.03] *^◊^*	0.02 [0.02;0.03]	0.02 [0.01;0.02]	0.03	0.02	0.051

Values are mean ± standard deviation (SD) or median [interquartile range], unless otherwise indicated. ^◊^ *p* < 0.05 vs. STEMI.

## Data Availability

Data collected in the study will be made available using the data repository Zenodo (https://zenodo.org/ accessed on 18 January 2022) with restricted access upon request to direzione.scientifica@ccfm.it.

## Supplementary Materials

The following supporting information can be downloaded at: https://www.mdpi.com/article/10.3390/ijms23031136/s1.
