# Supplementary material for: An Optimized MRM-Based Workflow of the l-Arginine/Nitric Oxide Pathway Metabolites Revealed Disease- and Sex-Related Differences in the Cardiovascular Field
Source: Int J Mol Sci. 2022 Jan 20;23(3):1136. doi: 10.3390/ijms23031136 (PMC8835333; doi:10.3390/ijms23031136)
Supplement: Supplementary file 1 [file ijms-23-01136-s001.zip › ijms-1563541-supplementary.pdf]

An optimized MRM-based workflow of the L-arginine/nitric oxide pathway metabolites revealed disease- and sex-related differences in the cardiovascular field

Benedetta Porro, Sonia Eligini, Edoardo Conte, Nicola Cosentino, Nicolò Capra, Viviana Cavalca, Cristina Banfi

SUPPLEMENTARY MATERIAL

TITLE	PAGE
**Table S1.** CCTA and OCT Characteristics of the Study Population	2
**Table S2.** Imprecision, LLOQ and LOD of the LC-MS/MS method	3
**Table S3.** Matrix effect (ME), recovery (ER) and process efficiency (PE) data for HArg in plasma	3
**Table S4.** Stability of HArg in plasma	3
HArg method validation procedures	4
**Table S5.** Mobile phase gradient	5
References	6

Table S1. CCTA and OCT Characteristics of the Study Population.

	CCTA group (*n* = 90)	no-CAD (*n* = 33)	nonob-CAD (*n* = 25)	ob-CAD (*n* = 32)	STEMI (*n*=14)	p-value
CCTA characteristics						
Total plaque volume, mm^3	88.5±128.2	-	99.6±126.7*	170.7±139.1$^{*,\circ}$	-	*<0.001*
Non-calcified plaque volume, mm^3	25.2±40.7	-	25.9±35.6*	50.7±49.3$^{*,\circ}$	-	*<0.001*
High risk plaque features >2, *n* (%)	26 (28.9)	0	6 (24.0)	20 (62.5)$^{*,\circ}$	-	*<0.001*
Non-calcified plaque volume HQ, *n* (%)	23 (25.5)	0	6 (24.0)	17 (53.1)$^{*,\circ}$	-	*<0.001*
Total plaque volume HQ, *n* (%)	23 (25.5)	0	5 (20.0)	18 (56.2)$^{*,\circ}$	-	*<0.001*
OCT characteristics						
Plaque erosion, *n* (%)	-	-	-	-	4 (28.6)	-
Plaque rupture, *n* (%)	-	-	-	-	8 (57.1)	-
Thrombus, *n* (%)	-	-	-	-	12(85.7)	-
Macrophage infiltration, *n* (%)	-	-	-	-	10 (71.4)	-
Macrophage NSD	-	-	-	-	3.8±2.5	-
Lipid plaque, *n* (%)	-	-	-	-	9 (64.3)	-
Fibrous plaque, *n* (%)	-	-	-	-	8 (57.1)	-
Calcific plaque, *n* (%)	-	-	-	-	1 (7.1)	-
MLA, mm^2	-	-	-	-	1.8±1.0	-
minimal cap thickness, μm	-	-	-	-	67.9±18.1	-

Values are mean ± standard deviation or median [interquartile range] or number (%), unless otherwise indicated. *p<0.05 *vs* no-CAD; °p<0.05 *vs* nonob-CAD; ◊p<0.05 *vs* STEMI. CCTA = Coronary Computed Tomography Angiography; MLA= minimal lumen area; NSD=normalized standard deviation.

Table S2. Imprecision, LLOQ and LOD of the LC-MS/MS method.

		Intrassay imprecision	Interassay imprecision	LLOQ (μM)	LOD (μM)
	Nominal concentration (μM), mean (SD)	CV, %	CV, %		
QC Low	0.46 (0.03)	7.5	3.8		
QC Medium	2.57 (0.17)	6.6	2.4	0.078	0.039
QC High	12.1 (0.41)	3.4	0.5		

LLOQ = Lower Limit Of Quantification; LOD = Limit Of Detection; QC = Quality Control; SD= Standard Deviation; CV = Coefficient of Variation.

Table S3. Matrix effect, extraction recovery and process efficiency data for HArg in human plasma.

	Mean Peak Area Ratio					
Concentration spiked (μM)	Set A	Set B	Set C	ME (%)	ER (%)	PE (%)
0.156	0.044	0.050	0.044	112.8	86.1	99.1
1.25	0.34	0.35	0.38	104.3	109.5	114.1
10	2.65	2.77	2.74	104.5	100.0	103.4

ME = matrix effect; ER = extraction recovery; PE = process efficiency.

Table S4. Stability of HArg in human plasma.

Temperature	Time					
	4 h	24 h	3 days	5 days	7 days	1 month
-20°C	-	99.5	91.2	86.2	84.1	91.7
+4°C	83.5	81.6	78.8	78.7	77.8	-
RT	76.8	72.5	69.7	68.4	67.9	-
Freeze-thaw			91.0			

RT = Room Temperature.

HArg method validation procedures

Ten human plasma samples were pooled together (pooled plasma, **PP**), aliquoted and used in the assay performance evaluation following the U.S. Food and Drug Administration guidelines [1, 2].

Due to the lack of a "blank" plasma sample, the assessment of assay imprecision was checked with quality controls (**QC**) prepared using PP: low concentration QC was made by diluting PP 1:20 v/v with NaCl 0.9%, PP as it is was used as middle QC and high QC was prepared by fortifying the endogenous HArg PP concentration with 1 mM standard solution (final concentration 10 μM). The intra-assay imprecision was determined by assaying 9 separate aliquots of each QC sample in a single batch. Inter-assay imprecision was determined by testing a single aliquot of each QC in 3 consecutive days.

The linearity and range of the calibration curve were evaluated with 8 standard calibrators over the concentration range 0.078-10 μM. Each calibrator was spiked with internal standard 13C715N4-HArg (final concentration 2 μM). Linearity of the assay was assessed by repeated analysis (*n*=3) of calibrators and linear regression analysis was used to determine the slope, intercept, and correlation coefficient (r^2).

The lower limit of quantification (**LLOQ**) was calculated as the lowest concentration providing a coefficient of variation (**CV**) <20% and an accuracy between 80% and 120%. The limit of detection (**LOD**) was defined as the lowest concentration that gave a signal-to-noise of at least 3.

The stability of the analyte at different temperatures was tested by analysing PP aliquots kept at -20°C, +4°C and +21°C for 4 h, 24h, 3 days, 5 days, 7 days and 1 month.

For the freeze/thaw stability study, two aliquots of PP were used. The first aliquot was immediately analysed for the quantification while the second one was frozen at -20°C and assayed after being freeze/thawed three times in three consecutive days.

Relative matrix effect (**ME**), extraction recovery (**ER**), and process efficiency (**PE**) and sample stability were evaluated according to Matuszewski et al[3]. All these parameters were assessed at three different concentrations of HArg (0.156, 1.25 and 10 μM final concentration) and the analysis was repeated 3 different times. Three sets of each concentration levels were prepared as follows: neat HArg standard solution (**set A**); PP samples spiked with HArg standard after matrix extraction (**set B**); PP samples spiked with HArg standard before matrix extraction (**set C**). The values of set A, B and C are expressed, in arbitrary units, as mean of area ratio. For set B and C the spiked area ratio of HArg was calculated by subtracting the basal endogenous HArg value (unspiked) from the measured area ratio. The rates of ME, RE and the overall PE were determined at each concentration tested as follows: ME (%) = peak area ratio from set B/peak area ratio from set A × 100; ER (%) = peak area ratio from set C/peak area ratio from set B × 100; PE (%) = peak area ratio from set C/peak area ratio from set A × 100.

Table S5. Mobile phase gradient.

Time (min)	% Solvent A (H_2O)	% Solvent B (CH_3CN)	% Solvent (NH_4COOH 15 mM pH 3.2)	% Solvent D (CH_3OH)
0	0	89.5	10	0.5
1	0	89.5	10	0.5
7	60	29.5	10	0.5
9	84.5	5	10	0.5
14	84.5	5	10	0.5
16	0	89.5	10	0.5
25	0	89.5	10	0.5

References

1. U.D.o.H.a.H.S. Food and Drug Administration, F., Center for Drug Evaluation and Research, *Guidance for Industry: Bioanalytical Method Validation, http://www.fda.gov/downloads/drugs/guidancecomplianceregulatoryinformation/guidances/ucm368107.pdf*. 2013.
2. Services, U. S. D. o. H. a. H.; Administration, F. a. D.; (CDER), C. f. D. E. a. R.; (CVM), C. f. V. M., Bioanalytical Method Validation Guidance for Industry. **2018**.
3. Matuszewski, B. K.; Constanzer, M. L.; Chavez-Eng, C. M., Strategies for the assessment of matrix effect in quantitative bioanalytical methods based on HPLC-MS/MS. *Analytical chemistry* **2003,** 75, (13), 3019-30.
